# Trends in Hospitalization and Inpatient Outcomes of Behçet's Disease: A Nationwide Inpatient Sample Study

**DOI:** 10.7759/cureus.7470

**Published:** 2020-03-30

**Authors:** Srikanth Naramala, Venu Madhav Konala, Sreedhar Adapa, Vijay Gayam, Jasdeep Sidhu, Sharmi Biswas, Mamtha Balla, Ganesh Prasad Merugu, Debendra Pattanaik

**Affiliations:** 1 Rheumatology, Adventist Medical Center, Hanford, USA; 2 Hematology and Oncology, Ashland Bellefonte Cancer Center, Ashland, USA; 3 Hematology and Oncology, King's Daughters Medical Center, Ashland, USA; 4 Nephrology, Kaweah Delta Medical Center, Visalia, USA; 5 Internal Medicine, Interfaith Medical Center, New York, USA; 6 Pediatrics, Weill Cornell Medicine, New York, USA; 7 Internal Medicine, ProMedica Toledo Hospital, Toledo, USA; 8 Internal Medicine, University of Toledo, Toledo, USA; 9 Geriatrics Medicine, The University of Toledo, Toledo, USA; 10 Rheumatology, The University of Tennessee Health Science Center, Memphis, USA

**Keywords:** behcet’s disease, behcet’s syndrome, cerebral venous thrombosis, vasculitis, orogenital ulcers, uveitis

## Abstract

Objective

After an extensive review of the literature, we discovered that no study had addressed trends in hospitalization for people with Behçet's disease (BD). Hence, in this study, we explore multiple variables in patients with BD in the US for the year 2016.

Methods

We analyzed the data relating to hospitalized patients for the year 2016 using the National Inpatient Sample (NIS) database with a listed discharge diagnosis of BD based on the International Classification of Diseases, 10th Revision (ICD-10) diagnosis code M35.2. The mean age in years, alive discharges, lumbar puncture procedures, type of hospital, the Charlson Comorbidity Index (CCI), comorbidities, mean length of stay (LOS) and factors affecting it, and total cost and charges for the admissions were analyzed. A p-value of <.05 was considered statistically significant.

Results

A total of 2,605 discharges with the diagnoses of BD were identified among 35.7 million overall discharges in 2016. Among patients hospitalized with underlying BD, the majority were white and female. The mean hospital LOS was 5.57 ± 0.37 days, which is higher than in the general population and statistically significant (5.57 days vs 4.62 days; p: 0.009). Mean LOS in patients undergoing lumbar puncture was 8.54 ± 2.91 days. Patients with BD had lower medical comorbidity burden (16.9% with a CCI of ≥3) vs the general population (24.67% with a CCI of ≥3) (p: 0.00). Medical comorbidities with a statistically significant difference in their prevalence in the two groups were renal disease, dementia, peptic ulcer disease, heart failure, rheumatologic disorders, malignancy, and dyslipidemia.

Conclusion

Increased awareness about this rare condition in an inpatient setting will help in the early identification of the disease and associated complications. This will help caregivers to provide quality care in a timely manner, thereby decreasing the morbidity, mortality, LOS, and hospital costs associated with BD.

## Introduction

Behçet's syndrome, also called Behçet's disease (BD), is a rare inflammatory condition with multiple systemic manifestations [1]. The exact cause of BD is unknown. In the US and Europe, estimates of prevalence have ranged from 0.12 to 7.5 per 100,000 people [2]. The common presentation includes the involvement of mucous membranes causing oral and genital ulcers and inflammation in the eyes (uveitis, retinal vasculitis). Other manifestations include acne-like lesions, erythema nodosum, cellulitis mimics, superficial thrombophlebitis, arthritis, and vasculitis of various organ systems.

Background/purpose

Our research showed that there was a paucity of data relating to nationwide trends in hospitalizations secondary to BD, leading us to analyze those trends in the current study. This study aims to evaluate the prevalence of BD and other commonly associated comorbid conditions and complications associated with it using the National Inpatient Sample (NIS) for the year 2016. The analysis includes examining the correlation of different aspects such as disease prevalence, age, race, sex, hospital region, hospital size, institution type, mortality, length of stay (LOS) in the hospital, and commonly associated comorbidities among the study population.

## Materials and methods

Data source

We utilized the NIS, which is a part of the healthcare cost and utilization project. It is the most extensive inpatient healthcare database that is publicly available. It contains all-payer data from 20% of nonfederal US acute care hospitals and is composed of more than seven million unweighted discharges per year. Each hospitalization can be transformed into weighted count by discharge weight provided in the dataset to yield national estimates. The data include discharge-level records based on demographics, diagnosis, procedures, healthcare statistics, LOS, and utilization data. The database provides hospitalization characteristics in a manner of location and type that divides the hospitals into teaching and non-teaching. The NIS 2016 uses the International Classification of Diseases, 10th Revision, Clinical Modification (ICD-10-CM) coding system to collect 25 discharge diagnoses and 15 procedures on each hospitalization.

Patients and outcomes

Patient-level variables included were age, sex, race, median household income for the patient's Zip code (quartiles), insurance status, and comorbidity burden measured as the Charlson comorbidity index (CCI). Race/ethnicity was categorized as white, black, Hispanic, and others. Insurance status was categorized as Medicare, Medicaid, private insurance, and uninsured/other based on the primary payer listed on the discharge record. Hospital level variables studied were hospital location (rural/urban), teaching status, hospital region, and hospital bed size. The US Census Bureau classifies hospital regions into the Northeast, Midwest, South, and West.

The CCI is a method of categorizing comorbidities of patients based on the ICD diagnosis codes found in administrative data, such as the NIS database. Each comorbidity category has an associated weight (ranging from one to six), based on the adjusted risk of mortality or resource use and the sum of all the weights results in a single comorbidity score for a patient. A score of zero indicates that no comorbidities were found. The higher the score, the more likely the predicted outcome will result in mortality or higher resource use.

Statistical analysis

A retrospective cross-sectional analysis of discharge data from the NIS database for all admissions related to BD for the year 2016 was performed. The data analysis was done using Stata 15 software (StataCorp, College Station, TX). Patients with underlying BD were our study group, and all the hospitalizations during 2016 were considered as the control group. Statistical hypotheses were tested using a p-value of <.05 as the level of statistical significance for univariate and multivariate analyses. The categorical and dichotomous variables were stated in percentages, and mean was reported for continuous variables (age, LOS, total cost, and total charge). A bivariate analysis was conducted to compare the patient demographics and other patient/hospital-related factors between the study and control groups.

Using the NIS, the trends in hospitalizations with a discharge diagnosis of BD in 2016 were studied. Analyses were performed using hospital-level sampling weights to obtain US national estimates. The NIS was used to find total hospitalizations in the adult population of ≥18 years of age with a listed discharge diagnosis of BD based on ICD-10 diagnosis code M35.2. Multiple regression analysis via a weighted generalized linear model was used to identify factors that were independently associated with increased LOS and, therefore, higher healthcare resource utilization. Further subgroup analyses were then performed to identify which of these factors could be contributing to increasing costs/LOS.

## Results

A total of 2,605 discharges with the diagnoses of BD were identified among the overall 35.7 million discharges in 2016. The patient demographic details are summarized in Table 1. Among patients hospitalized with underlying BD, 70.8% were females as compared to 55.3% in overall admissions (p: 0.00). The mean age for BD patients was 48.10 ± 0.81 as compared to 49.00 ± 0.19 in all hospital admissions, and it was found to be not statistically significant (p: 0.67). Most patients were whites (74.45%), followed by blacks (12.17%), and Hispanics (8.11%) in our study group. On the other hand, 65.39% of the patients were whites, 15.20% blacks, and 12.27% Hispanics in all hospital admissions (p: 0.00). Most of the hospitalizations in the study population were noted in urban setting (95.05% vs 90.66% overall, p: 0.02), large hospitals (56.43% vs 52.26% overall, p: 0.16), and non-teaching hospital settings (73.32% vs 65.40% overall, p: 0.00). Most of the admissions in our study population were in the Southern United States (35.5% vs 39.30% overall, p: 0.02); 39% of these patients had Medicare as primary insurance provider followed by private insurance (37. 49%), whereas 40.97% of patients had Medicare and 31.11% had private insurance overall. These differences were statistically significant (p: 0.01). The majority of patients in the study population belonged to groups with higher median income as compared to the overall population (p: 0.00) and were from small metropolitan areas with <1 million population as compared to overall admissions, which reported most patients from micropolitan areas and non-urban areas (p: 0.10).

**Table 1:** Patient demographics SD: standard deviation; COPD: chronic obstructive pulmonary disease

Variable	Behçet's disease patients	All hospital admissions	P-Value
Total	2,605	35.7 million	
Female, %	70.77	55.31	0
Age in years, mean ± SD	48.10 ± 0.81	49.00 ± 0.19	0.67
Discharged alive, %	98.85	98	0.29
Lumbar puncture, %	3.8	0.74	0
Race, %			0
White	74.45	65.39	
Black	12.17	15.2	
Hispanic	8.11	12.27	
Asian/Pacific Islander	2.23	3.07	
Native American	0.2	0.66	
Other	2.84	3.41	
Weekend admissions, %	20.15	20.34	0.92
Insurance provider, %			0.01
Medicare	39	40.97	
Medicaid	21.43	23.92	
Private	37.49	31.11	
No insurance	2.17	4	
Charlson Comorbidity Index, %			0
0	40.69	45.3	
1	26.1	17.55	
2	16.31	12.48	
≥3	16.9	24.67	
Median income in patient's Zip code, %			0
$1–42,999	21.87	30.7	
$43,000–53,999	26.04	25.42	
$54,000–70,999	25.45	23.92	
≥$71,000	26.64	19.96	
Patient residence, %			0.1
Large metropolitan areas with at least 1 million residents	31.4	29.93	
Small metropolitan areas with less than 1 million residents	48.75	23.85	
Micropolitan areas + non-urban	19.85	46.22	
Hospital region, %			0.02
Northeast	21.5	18.5	
Midwest	18.81	22.23	
South	35.5	39.3	
West	24.19	19.97	
Bed size, %			0.16
Small	15.74	18.71	
Medium	27.83	29.03	
Large	56.43	52.26	
Teaching hospital Status, %			0
Teaching	26.68	34.6	
Non-teaching	73.32	65.4	
Hospital location, %			0.02
Urban	94.05	90.66	
Non-urban	5.95	9.34	
Comorbidities, %			
Diabetes	19	22.07	0.13
Peripheral vascular disease	5.37	6.54	0.28
COPD	22.46	19.56	0.13
Renal disease	9.02	13.63	0
Liver disease	4.99	4.17	0.41
Cerebrovascular disease	6.14	6.57	0.73
Myocardial infarction	5.18	6.93	0.12
Dementia	1.15	5.44	0
Peptic ulcer disease	3.45	1.28	0
Congestive heart failure	8.67	14.44	0
Rheumatoid disease	13.43	2.29	0
HIV disease	0.19	0.34	0.57
Malignancy	3.26	0.74	0
Smoking	17.27	21.55	0.5
Alcoholism	0.38	0.95	0.19
Hypertension	36.08	32.37	0.1
Dyslipidemia	20.53	25.69	0.01
Pulmonary hypertension	0	0.03	0.8

Patients admitted with underlying BD had lower medical comorbidity burden (16.9% with a CCI of ≥3) vs ≥3s general population (24.67% with a CCI of ≥3) (p: 0.00). Medical comorbidity with statistically significant difference in their prevalence in the two groups were renal disease (9.02% vs 13.63%, p: 0.00), dementia (1.15% vs 5.44%, p: 0.00), peptic ulcer disease (3.45% vs 1.28%, p: 0.00), congestive heart failure (8.67% vs 14.44%, p: 0.00), rheumatologic disorders (13.43% vs 2.29%, p: 0.00), malignancy (3.26% vs 2.74%, p: 0.00) and dyslipidemia (20.53% vs 25.69%, p: 0.01).

Lumbar puncture was performed more in patients with BD as compared to the overall patient population (3.8% vs 0.74%, p: 0.00). The factors affecting LOS in BD patients are summarized in Table 2. The mean LOS in BD patients was 5.57 ± 0.37 days. Mean LOS in patients among study populations undergoing lumbar puncture was 8.54 ± 2.91 days. Lumbar puncture was found not to affect LOS in a statistically significant manner.

**Table 2:** Factors affecting hospital length of stay in patients with underlying Behçet's disease COPD: chronic obstructive pulmonary disease

Variable	Coefficient/adjusted mean difference/beta	95% confidence interval	P-value
Female sex	-1.49	(-3.31)–0.33	0.11
Age	0.67	(-0.08)–1.44	0.8
Race			
White	Reference		
Black	1.2	(-0.99)–3.40	0.28
Hispanic	3.09	(-1.74)–7.93	0.21
Asian/Pacific Islander	0.002	(-2.54)–2.54	0.99
Native American	4.82	4.03–5.60	0
Other	2.25	(-1.84)–6.34	0.28
Weekend admission	-0.35	(-1.55)–0.85	0.57
Insurance			
Medicare	Reference		
Medicaid	1.54	(-1.13)–4.22	0.26
Private	-0.11	(-1.33)–1.11	0.86
No insurance	-1.66	(-3.42)–0.12	0.07
Income			
$1–42,999	Reference		
$43,000–53,999	-3.2	(-5.26)–(-1.18)	0
$54,000–70,999	-1.77	(-3.76)–0.21	0.08
≥$71,000	-0.44	(-3.23)–2.35	0.76
Patient residence			
Large metropolitan areas with at least 1 million residents	Reference		
Small metropolitan areas with less than 1 million residents	-0.41	(-1.91)–1.10	0.59
Micropolitan areas + non-urban	0.66	(-1.32)–2.64	0.51
Hospital location			
Rural	Reference		
Urban	1.08	(-0.83)–2.99	0.27
Charlson Comorbidity Index			
0	Reference		
1	1.2	(-0.83)–3.23	0.25
2	2.3	(-0.99)–5.60	0.17
≥3	7.52	2.81–12.21	0
Bed size			
Small	Reference		
Medium	-0.33	(-1.56)–0.90	0.59
Large	2.04	0.75–3.34	0
Hospital teaching status			
Non-teaching	Reference		
Teaching	0.58	(-0.61)–1.77	0.34
Hospital region			
Northeast	Reference		
Midwest	0.36	(-1.48)–2.21	0.7
South	0.38	(-0.94)–1.72	0.57
West	1.3	(-1.18)–3.79	0.31
Lumbar puncture	3.09	(-2.66)–8.85	0.29
Comorbidities			
Diabetes	-1.11	(-3.43)–1.22	0.35
Peripheral vascular disease	-2.19	(-4.65)–1.19	0.43
COPD	-0.64	(-3.22)–1.94	0.63
Renal disease	-3.14	(-6.55)–0.27	0.07
Liver disease	0.44	(-4.95)–5.83	0.87
Cerebrovascular disease	3.43	(-2.08)–8.95	0.22
Myocardial infarction	-1.25	(-3.49)–0.98	0.27
Dementia	-0.61	(-4.28)–3.05	0.74
Peptic ulcer disease	No observations		
Congestive heart failure	-0.79	(-2.76)–1.19	0.43
Rheumatoid disease	-0.85	(-3.54)–1.84	0.54
HIV disease	0.002	(-0.001)–0.006	0.32
Malignancy	1.05	(-8.24)–10.34	10.34
Smoking	-2.89	(-5.28)–0.50	0.02
Alcoholism	0.003	(-0.001)–0.009	0.16
Hypertension	-1.56	(-2.92)–(-0.20)	0.02
Dyslipidemia	-0.58	(-1.77)–0.61	0.34
Pulmonary hypertension	No observations		

Native Americans had significantly higher LOS compared to white Americans (adjusted mean difference: 4.82 days, p: 0.00). Patients in the median income group of $42,000-53,999 had lesser LOS compared to the lowest income stratum with a median income of <$42000 (adjusted mean difference: -3.22 days, p: 0.00). Patients with higher comorbidity burden (CCI of ≥3) were noted to have significantly longer LOS as well (adjusted mean difference: 7.52 days, p: 0.00). Patients admitted to large hospitals were also found to have significantly longer LOS compared to small hospitals (adjusted mean difference: 2.04 days, p: 0.00). Except for hypertension (adjusted mean difference: -1.56 days, p: 0.02) and smoking (adjusted mean difference: -2.89 days, p: 0.02), other medical comorbidities affecting the LOS in our study population were not found to be statistically significant.

The 10 most common admission diagnoses were identified with ICD-10-CM codes in our study population; these are sepsis (7.86%), pneumonia (2.69%), left upper limb cellulitis (1.34%), unspecified gastrointestinal bleeding (1.34%), clostridium difficile colitis (1.15%), left lower limb cellulitis (1.15%), acute exacerbation of chronic obstructive pulmonary disease (COPD) (1.15%), urinary tract infection (UTI) (0.8%), left hip osteoarthritis (0.8%), and aspiration pneumonitis (0.8%). All the above findings are summarized in Table 3.

**Table 3:** Most common hospital admission diagnoses for patients with Behçet's disease ICD 10: International Classification of Diseases, 10th Revision; COPD: chronic obstructive pulmonary disease

Sr. no.	Diagnosis	ICD 10 Code	Total cases, n (%)
1	Sepsis	A41.9	205 (7.86)
2	Pneumonia	J18.9	70 (2.69)
3	Left upper limb cellulitis	L03.114	35 (1.34)
4	unspecified gastrointestinal bleeding	K92.2	35 (1.34)
5.	Clostridium difficile colitis	A04.7	30 (1.15)
6.	Left lower limb cellulitis	L03.116	30 (1.15)
7.	Acute COPD exacerbation	J44.1	30 (1.15)
8.	Unspecified urinary tract infection	N39.0	20 (0.8)
9.	Left hip osteoarthritis	M16.12	20 (0.8)
10.	Aspiration pneumonitis	J69.0	20 (0.8)

Out of the 2,605 patients admitted with BD, 2,575 patients (98.85%) were discharged alive as compared to 98% alive discharges overall (p: 0.29). Resource utilization details for BD patients are summarized in Table 4. Costs reflect the actual expenses incurred in the production of hospital services, such as wages, supplies, and utility costs; charges represent the amount a hospital billed for the case. The mean cost of hospitalization for patients with BD was $14,427, and the mean hospital charge for each admission was $58,772.31. The mean cost and charge for BD patients undergoing lumbar puncture were $27,261 and $97,649 respectively. However, these differences were found not statistically significant. Total Cost and the total charge for all the admissions with underlying BD were $37.6 million and $153 million respectively.

**Table 4:** Resource utilization in Behçet's disease patients SD: standard deviation: LOS: length of stay *Costs reflect the actual expenses incurred in the production of hospital services, such as wages, supplies, and utility costs **Charges represent the amount a hospital billed for the case

	Patients undergoing lumbar puncture	Patients not undergoing lumbar puncture	P-Value
Total number	100	2,505	
LOS, days, mean ± SD	8.54 ± 2.91	5.57 ± 0.37	0.29
Mean total cost*, $	27,261	14,427.98	0.09
Mean total charge**, $	97,649	58,772.31	0.15
Total cost*, $	2.7 million	37.6 million	
Total charge**, $	9.7 million	153 million	
Total LOS, days	854	13,952.85	

## Discussion

BD is a multisystem disorder of unknown etiology characterized by skin and mucosa lesions, arthritis, ocular issues, pulmonary involvement, vasculitis, thrombosis, and gastrointestinal and neurological involvement [1]. Recent literature mentions both autoinflammatory and autoimmune factors playing a role in the development of BD. Autoinflammatory disorders are various conditions manifested secondary to dysfunction of the innate immune system. They lead to the inappropriate elevation of cytokines without any triggering events typically presenting as recurrent self-limited exacerbations, along with remissions happening at different intervals. Human leukocyte antigen (HLA)- B51 is the allele predominantly associated with BD, which is related to autoimmune diseases [3]. Dysregulation of the humoral (adaptive) immune system in autoimmune diseases causes the inflammatory processes. Strong genetic background is reported in BD, and several environmental factors can trigger the manifestations of BD in genetically susceptible individuals [1,3]. The Silk Route (the Mediterranean region to the Middle East and the Far East) is the geographical area where BD is most prevalent. The frequency of the HLA-B51 allele related to BD is higher among the population living in this region [4].

The mean age of onset of BD is around the third decade of life. The mortality rate with BD is higher among patients younger than 35 years of age. Male sex is another important factor of mortality in BD due to the predominance of large arterial involvement and neurologic involvement in males. Almost every systemic BD manifestation is more severe in males compared to females [5].

There is no specific diagnostic test is available to detect BD; it is mainly a clinical diagnosis, and the International Study Group (ISG) diagnostic criteria are useful. According to the ISG criteria, BD can be detected based on the presence of recurrent oral ulceration along with any two of the following conditions/factors: skin lesions, positive pathergy test, ocular findings, and recurrent genital ulceration. The disease course is characterized by frequent relapses and remissions [6].

**Table 5:** International Study Group criteria for the diagnosis of Behçet's disease According to the ISG criteria, BD can be detected based on the presence of the first condition in the table (recurrent oral ulceration) along with any two of the following four conditions/factors in the table: skin lesions, a positive pathergy test, ocular findings, and recurrent genital ulceration. These findings need to be taken into consideration only in the absence of other clinical explanation

Condition/factor	Description
Recurrent oral ulceration	Recurrent major or minor aphthous or herpetiform lesions noticed by the patient or physician at least three times in one-year period
Skin lesions	Papulopustular lesions or pseudofolliculitis, erythema nodosum, or acneiform nodules observed by the physician in post-adolescent patients not on steroids
Positive pathergy test	Read by the physician at 24–48 hours
Ocular findings	Uveitis (anterior or posterior), slit lamp showing cells in the vitreous or retinal vasculitis seen by an ophthalmologist
Recurrent genital ulceration	Aphthous ulceration or scarring noticed by the patient or physician

Pathergy testing constitutes provocative skin testing with a prick by a 20-gauge needle, which results in erythematous papulopustular response 24-48 hours after the injury. BD can involve multiple systems. Here we discuss the various systemic manifestations of BD based on the findings of our study.

Orogenital ulceration

The hallmark of BD is recurrent oral aphthae. Ulcers can be very small (<10 mm) to large (>10 mm) and can be herpetiform. Usually, ulcers arise on the buccal mucosa and the inner lip mucosa, but tongue, palate, and pharynx can also be affected. Ulcers are painful and can leave a scar and have an erythematous halo around them. BD oral ulcers can resolve spontaneously within 10-14 days; though occasionally new ulcers arise, leading patients to think they are sustained.

Genital ulcers are less frequent than oral ulcers. They are also painful and have a higher chance of scarring compared to oral ulcers. In females, vulva, vagina, and cervix can be affected; in males, ulcers are more common on the scrotum. Penile ulcers in men should be suspected to have associations with HLA-B27-related spondylarthritis. All the non-healing oral and genital ulcers should be biopsied to exclude other etiologies [7,8]. Other cutaneous lesions are also common in BD, in the forms of papules and pustules resembling acne. Subcutaneous nodules and erythema nodosum are other skin manifestations of BD [8]. In our study, dermatological presentations such as erythema nodosum and recurrent oral and genital ulceration could not be evaluated in BD as these are primarily diagnosed and treated on an outpatient basis, which is one of the limitations of the study. Left upper limb cellulitis (1.34%) and left lower limb cellulitis (1.15%) were among the ten most common admission diagnosis in our study population, which should be considered high on the differential; and there is a high possibility of misdiagnosis considering less awareness of different dermatological presentations of BD in the inpatient setting. Another limitation is that our dataset is an administrative database, which lacks access to the patients' charts to get detailed information on how these cases were managed and the response to treatment, which would have given a better understanding of whether it was true cellulitis or a manifestation of BD.

Ocular inflammation

In 10-15% of the patients with BD, ocular symptoms arise within two-three years of diagnosis. Ocular involvement is common in BD and severe in males and individuals younger than 30 years of age. In general, ocular symptoms arise followed by recurrent oral ulcers; but in 20% of the patients, an ocular presentation can be the earliest manifestation [8]. Bilateral eyes can be involved, and the inflammation is recurrent in nature. Pan uveitis, anterior uveitis, posterior uveitis, retinal vasculitis, optic neuritis, and retinal vein occlusion are the different forms of ocular presentations that can cause significant morbidity. Posterior uveitis with poor visual acuity indicates poor prognosis in 39% of the patients [9]. No patients with underlying BD were noted to have non-infectious uveitis or retinal vasculitis in our study (again, mainly outpatient diagnoses and very few ophthalmologic emergencies).

Neurologic involvement

Headache is the most common neurological feature in BD. Vascular involvement is the cause of the headache in the majority of patients; it resembles migraine and comes with aura. Tension headache, analgesic associated headache, and chronic daily headache can also happen. Sometimes, the severity of headache correlates with the disease progression. In imaging, no structural lesion can be usually found. Patients respond well to antimigraine therapy [9,10]. Neuro-Behçet syndrome (neurological complications of Behçet’s syndrome excluding headache) occurs in 12 % of the cases, often after a few years of other systemic features. The parenchymal disease is more common than neurovascular type, and both types are more common in males than females. The peripheral nervous system rarely gets affected. There are some reports of large fiber neuropathies, acute polyradiculopathies, and mononeuritis multiplex. Severe myositis in children and isolated cranial neuropathies followed by concurrent meningitis are some of the manifestations of peripheral nervous system BD [11]; 20% of patients with BD suffered from neurovascular diseases. Dural sinus thrombosis and intracranial and extracranial aneurysm are the most common symptoms. Prognosis of neurovascular BD is good if the patients survive the initial event. Peripheral nervous system BD prognosis is generally uncertain [10,11]. We found very few patients having any neurological involvement. Correlation between headache/migraine and BD was not evaluated since there were no admissions with a diagnosis of headache/migraine (treated mainly as an outpatient or in the emergency room).

Psychiatric and cognitive dysfunction

In BD, cognitive dysfunction is common in the form of working memory deficit, frontal executive dysfunction, and attention deficit. These symptoms are severe in patients with neurological involvement. Anxiety and depression are also very common in patients with BD. Recent studies have found that behavioral changes are common in BD; however, major psychiatric symptoms are rare [10,11]. There are some chronic, multifocal, and subcortical changes in the white matter found on MRI [12]. Dementia was seen in 1.15% of the cases with BD compared to 5.44% in the control group, and this difference was statistically significant with a p-value of 0.00.

Pulmonary involvement

Around 5% of the patients with BD have pulmonary involvement. A wide range of abnormalities can manifest as a part of BD involving the chest. A typical feature is a pulmonary artery aneurysm with or without thrombosis. Hemoptysis can be a predominant symptom due to aneurysm rupture or from in situ thrombosis. Pulmonary artery embolism, bronchiectasis, atelectasis, and subpleural nodules are the other reported features of pulmonary BD [13]. Relationship of BD with hematologic/vascular conditions such as cerebral venous thrombosis, intracranial arterial or venous thrombosis, pulmonary embolus, pulmonary aneurysms, and deep vein thrombosis (DVT) could not be assessed due to very low/zero patients having any of these conditions along with BD.

Musculoskeletal system

In BD, musculoskeletal symptoms are very common. Arthritis and arthralgia are the most prevalent musculoskeletal features in BD. Asymmetric arthritis mainly affects knee, ankles, and wrists but can also be seen in other joints as well. In many patients, fibromyalgia coexists with BD and is associated with anxiety and depression. Osteonecrosis and myalgia are some of the rare findings in BD [14]. In our study, fibromyalgia in BD could not be evaluated as it is mostly an outpatient diagnosis. Left hip osteoarthritis was seen in some patients with BD (0.8%); however, it was not statistically significant.

Constitutional features

Constitutional symptoms like fatigue is a universal feature in BD and have a huge impact on the patient’s quality of life. Overall, BD compromises the quality of life in adult patients due to fatigue, pain/discomfort, and difficulties with mobility and usual daily activities [15].

## Conclusions

The higher prevalence of the diagnosis of BD in urban settings may be associated with the increased availability of specialists (rheumatologists, dermatologists, ophthalmologists, and neurologists) in the inpatient setting. In this study, we analyzed the prevalence of BD and its associated systemic manifestations, common associations with other diagnoses/diseases during the admission, related LOS, clinical outcomes, and total hospitalization costs and charges. Increased awareness about this rare condition in the inpatient setting may help in early identification of the disease and associated complications. This will enable the caregivers to provide quality care in a prompt manner, thereby decreasing the morbidity, mortality, LOS, and hospital costs. Further studies should be conducted to understand the association between BD and other rheumatologic conditions and also the most common comorbid conditions that are seen with BD.
